# Phenotypic features and genetic characterization of male breast cancer families: identification of two recurrent BRCA2 mutations in north-east of Italy

**DOI:** 10.1186/1471-2407-6-156

**Published:** 2006-06-09

**Authors:** GianMaria Miolo, Lara Della Puppa, Manuela Santarosa, Clelia De Giacomi, Andrea Veronesi, Ettore Bidoli, Maria Grazia Tibiletti, Alessandra Viel, Riccardo Dolcetti

**Affiliations:** 1 Division of Experimental Oncology 1, Centro di Riferimento Oncologico, National Cancer Institute, Aviano, Italy; 2 Division of Medical Oncology C, Centro di Riferimento Oncologico, National Cancer Institute, Aviano, Italy; 3 Epidemiology Unit, Centro di Riferimento Oncologico, National Cancer Institute, Aviano, Italy; 4 Pathology Unit, Insubria University, Varese, Italy; 5 Immunovirology and Biotherapy Unit, Centro di Riferimento Oncologico, National Cancer Institute, Aviano, Italy

## Abstract

**Background:**

Breast cancer in men is an infrequent occurrence, accounting for ~1% of all breast tumors with an incidence of about 1:100,000. The relative rarity of male breast cancer (MBC) limits our understanding of the epidemiologic, genetic and clinical features of this tumor.

**Methods:**

From 1997 to 2003, 10 MBC patients were referred to our Institute for genetic counselling and BRCA1/2 testing. Here we report on the genetic and phenotypic characterization of 10 families with MBC from the North East of Italy. In particular, we wished to assess the occurrence of specific cancer types in relatives of MBC probands in families with and without BRCA2 predisposing mutations. Moreover, families with recurrent BRCA2 mutations were also characterized by haplotype analysis using 5 BRCA2-linked dinucleotide repeat markers and 8 intragenic BRCA2 polymorphisms.

**Results:**

Two pathogenic mutations in the BRCA2 gene were observed: the 9106C>T (Q2960X) and the IVS16-2A>G (splicing) mutations, each in 2 cases. A BRCA1 mutation of uncertain significance 4590C>G (P1491A) was also observed. In families with BRCA2 mutations, female breast cancer was more frequent in the first and second-degree relatives compared to the families with wild type BRCA1/2 (31.9% *vs*. 8.0% p = 0.001). Reconstruction of the chromosome phasing in three families and the analysis of three isolated cases with the IVS16-2A>G BRCA2 mutation identified the same haplotype associated with MBC, supporting the possibility that this founder mutation previously detected in Slovenian families is also present in the North East of our Country. Moreover, analysis of one family with the 9106C>T BRCA2 mutation allowed the identification of common haplotypes for both microsatellite and intragenic polymorphisms segregating with the mutation. Three isolated cases with the same mutation shared the same intragenic polymorphisms and three 5' microsatellite markers, but showed a different haplotype for 3' markers, which were common to all three cases.

**Conclusion:**

The 9106C>T and the IVS16-2A>G mutations constitute recurrent BRCA2 mutations in MBC cases from the North-East of Italy and may be associated with a founder effect. Knowledge of these two recurrent BRCA2 mutations predisposing to MBC may facilitate the analyses aimed at the identification of mutation carriers in our geographic area.

## Background

Approximately 5–10% of breast and ovarian cancer cases are hereditary, occurring mainly in women with germ-line mutations in the BRCA1 or BRCA2 genes [1-4]. Although earlier estimates suggested that *BRCA1 *and *BRCA2 *mutations were responsible for 75% of breast cancer families [4-7] recent population-based studies indicate that these rates may have been overestimated. In fact, the percentage of high-risk families associated with *BRCA1 *and *BRCA2 *mutations seems to be around 25% in all groups investigated [8-12].

BRCA1 germ-line mutations are also associated with an increased risk of other cancer types, including those affecting colon, pancreas, stomach and fallopian tubes [13,14]. Other possible sites of cancer for BRCA1 mutation carriers included cutaneous melanoma, basal cell carcinoma and sarcoma [15]. Although BRCA1 mutations have been described also in men with breast cancer, the presence of germ-line mutations in the BRCA1 gene does not seem to convey a significantly increased risk for MBC [16]. However, this issue is still controversial, since a recent study carried out in 483 BRCA1 mutation carriers reported a 58-fold increased risk [13].

Available evidence indicates that BRCA2 mutation carriers have an increased risk of breast cancer in both males and females, as well as of prostate and pancreatic carcinomas. Carcinomas of the stomach, gallbladder bile ducts, cutaneous melanoma, and basal cell carcinoma are also slightly more prevalent in carriers of BRCA2 mutations [17,18]. The large majority of data accumulated so far indicates that, unlike what reported for female breast cancer (FBC), MBC is mainly linked to BRCA2 gene mutations. In fact, up to 30% of MBC were associated with BRCA2 mutations, whereas only less than 4% of the cases carried BRCA1 gene mutations, with variable percentages depending on founder mutations [19,20].

To date, only few studies have attempted to estimate the risk of breast cancer in male carriers of a BRCA2 mutation. The analysis of two large families carried out by Easton *et al*. [21] resulted in a cumulative risk of MBC of 6.3% (95% CI 1.4% to 25.6%) by age 70. In contrast, the study by Thompson et al. [17], based on 59 cases, failed to detect a significantly higher cumulative risk of MBC by 70 years of age (2.8%; 95% CI 0.6 – 13.0%). However, the risk increased to 6.9% (95% CI 1.2% to 38,6%) by 80 years, 80-fold higher compared to the general population. The present study was carried out with the aim to characterize the genetic and phenotypic features of families with MBC from the North East of Italy. In particular, we wished to assess the occurrence of specific cancer subtypes in first, second and third degree relatives of MBC probands in families with and without BRCA2 predisposing mutations. Moreover, we also searched for the presence of recurrent mutations in this area due to a possible founder effect.

## Methods

From 1997 to 2003, 197 patients with breast or ovarian cancer with a family history of these tumors were referred for genetic counselling to the Centro di Riferimento Oncologico (National Cancer Institute, Aviano, Italy). The present study is part of an Institutional program on hereditary tumors approved by local Ethical Committee. Families were selected according to previously reported criteria [22]. All consecutive male patients with a breast cancer, independently of age and family history, were included and counselled for genetic testing for BRCA1 and BRCA2 gene mutations. Ten families were ascertained by a male index case; one patient with MBC (BR175) also developed prostatic cancer at the age of 66 (Table 1). One additional male patient (BR331), showing an atypic intracystic papilloma of the breast together with a melanoma of the ear, was not included in the statistical analysis. All individuals tested for BRCA1/2 mutations gave a signed informed consent.

Blood samples were obtained from each proband and after the identification of a specific mutation the analyses were extended to 98 relatives. Screening for mutations in the BRCA1 and BRCA2 genes was carried out by a combination of protein truncation test, single strand conformation polymorphism, and sequencing techniques (Table 1). Primer sequences and PCR conditions were previously described [22,23]. All mutations and genetic variants were named according to Antonarakis et al. [24]. Missense mutations and mutations occurring within intronic regions whose clinical significance has not yet been reported were defined as genetic unclassified variants. Germline rearrangements of BRCA1 and BRCA2 gene were also analysed by multiplex ligation dependent probe amplification (MLPA).

The haplotype associated with the 9106C>T BRCA2 mutation was analyzed using five BRCA2-linked di-nucleotide repeat markers: D13S290, D13S260, D13S1698, D13S171, and D13S1695, whereas for the IVS16-2A>G mutation the D13S290, D13S171, and D13S1695 markers were used.

The first three markers were placed in 3' flanking of the BRCA2 gene whereas the other two were placed in the 5' flanking of the BRCA2 gene. The order of markers from centromere to telomere is as follows: cen-D13S290, D13S260, D13S1698, D13S171, D13S1695-tel. PCR products were analyzed on the ABI Prism 3100 using the POP4 matrix and fluorescently labelled primers. For these markers, a heterozygosity of 46%, 78%, 63%, 72%, and 79% has been reported in the Genome Data Base, respectively [25]. Alleles were numbered according to the size of each microsatellite repeat marker. The disease-associated haplotypes were deduced from allelic segregation in known carriers by inspection of segregating genotypes in the analyzed families. Moreover, for the haplotype analysis of the 9106C>T mutation, eight internal BRCA2 polymorphisms were analyzed: 203G>A (exon 2), IVS8+56C>T (intron 8), 1342A>C (exon 10), 3624A>G (exon 11), 4035T>C (exon 11), IVS11+80del4 (intron 11), 7470A>G (exon 14), IVS16-14T>C (intron 16). For the haplotype analysis only, besides the 4 families ascertained by a MBC index case, 6 additional families were also analyzed, two carrying of the 9106C>T mutation and 4 with the IVS16-2A>G mutation. All these families were ascertained by a FBC index case, showed a positive family history of breast and/or ovarian cancer and came from the same geographic area.

### Statistical analyses

The first and second degree relatives of the male index case of BRCA2 positive families were compared to first and second degree relatives of families without a BRCA2 mutation but with or without familial history for breast cancer, using chi-square analyses [26]. The family with a BRCA1 mutation with unclear significance was excluded from this comparison. Odds Ratios (OR), and their corresponding 95% Confidence Interval (CI), was computed using unconditional logistic regression model [27] in order to calculate the odds to develop a tumor in the first and second degree relatives conditional on the BRCA2 status of the proband.

## Results

The 10 MBC probands had a mean age at the time of diagnosis of 53.3 years (range 38–70 years). 3/10 patients were younger than 50 years, whereas 7 were ≥50 years. Only one patient (BR247) developed a breast cancer before the age of 40. Four of the 10 patients (40%) carried a predisposing mutation in the BRCA2 gene (Table 1). Five of the remaining six cases carried wild-type (w.t.) BRCA1 and BRCA2 genes whereas in one case (BR282) a missense BRCA1 mutation 4590C>G (P1491A), causing a Pro>Ala replacement in position 1491 with unclear significance was observed (Table 1). The family history of this latter case showed two second-degree female relatives affected by breast cancer, one belonging to the maternal side and the other to the paternal side. This mutation was not reported in the Breast Cancer Information Core database [28]. Although mutations in the BRCA1 gene do not seem to be associated with increased risk of MBC, further studies are required to assess the possible predisposing role of this mutation in case BR282. It was not possible to assess whether this variant was present in other affected relatives because none of them was alive.

The six cases negative for point mutations were also analyzed for BRCA1 and BRCA2 genes rearrangements by MLPA. None of these cases showed structural alterations of BRCA1 and BRCA2 genes. The median age of diagnosis was 54.5 years for the 4 BRCA2 mutation carriers and 53.8 years for the other cases, excluding the unclassified variant. The 4 BRCA2 positive cases carried only two distinct mutations: the 9106C>T, involving exon 22 and leading to a premature protein truncation (cases BR22 and BR175), and the IVS16-2A>G, involving intron 16 and leading to a partial or total skipping of exon 17 (cases BR195 and BR243) [29]. Both these mutations were localized outside the Ovarian Cluster Cancer Region and were previously reported also by others in the BIC database (three times for the 9106C>T mutation and four times for the IVS16-2A>G mutation in all). The carriers of the IVS16-2A>G mutation and those with the 9106C>T mutation showed a median age of breast cancer onset of 60 and 49 years, respectively.

In one IVS16-2A>G positive family, a first-degree relative (the father of the BR195 proband) was affected by a MBC diagnosed at 38 years. When this case was also included in the analysis, the median age of MBC onset in carriers of the IVS16-2A>G mutation decreased to 52.7 years.

In 8/10 cases the histologic characterization of the tumor was available. Invasive ductal carcinoma (75%) was the most frequent histologic subtype followed by intraductal carcinoma (25%). Another case, diagnosed as atypic intracystic papilloma, which showed also a melanoma of the ear, was found negative for BRCA1 and BRCA2 mutations. This case was not included in the statistical analysis. All the 6 assessable cases were oestrogen and progesterone receptors positive by immunohistochemistry (Table 1), confirming previous findings indicating that MBC have a higher rate of positivity to hormone receptors than do FBC (90% vs. 77%) [30]. However, when MBC were compared with cancers from postmenopausal women, the positivity rates were similar. All our BRCA2 positive cases showed a positive family history for breast and/or ovarian cancer (Table 1).

Two out of three cases of prostatic cancers were observed at 61 and 75 years, respectively, in w.t. families. In the four BRCA2 mutation carrier families, breast cancer was more frequent in the first and second-degree relatives than in the five w.t. families. In fact, as compared with w.t. families, in BRCA2 mutation carrier families there was a significant higher rate of affected first and second degree relatives (31.9% *vs*. 8.0% p = 0.001). These results indicate that first and second degree relatives of index cases with BRCA2 mutation carrier families have a five times greater risk to develop a breast cancer (Table 2). The two MBC cases without family history of cancer resulted negative for BRCA1/2 mutations. In three of the four families with a BRCA2 gene mutation, the molecular analysis was extended to ten non-affected relatives, (six males and four females), allowing the identification of 5 additional BRCA2 mutation carriers (2 males and 3 females).

**Figure 1:**
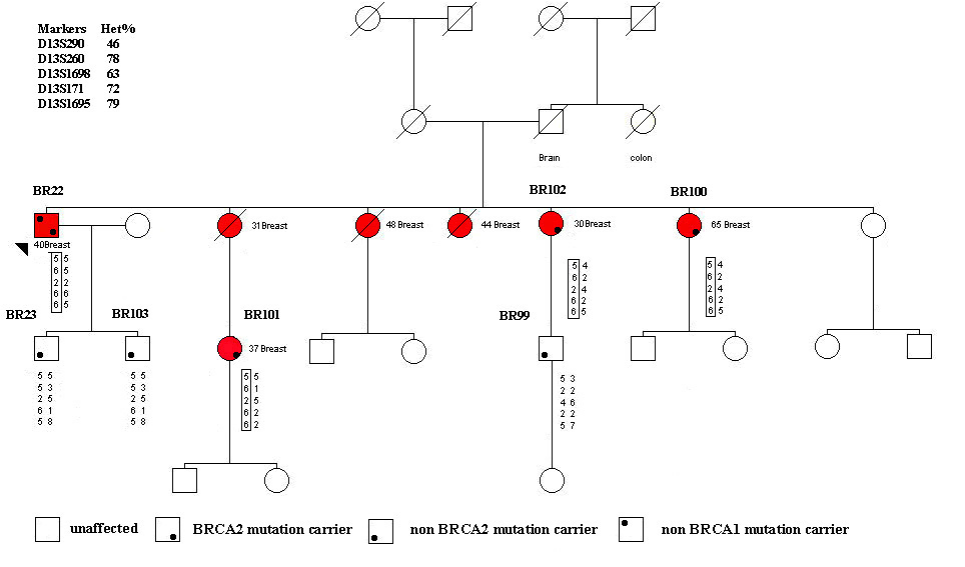
Pedigree of the family 25 with the reconstruction of the chromosome phasing. The type of cancer and age at diagnosis are indicated. Affected individuals are indicated by a red symbol and proband is denoted by arrow. The shared haplotype segregating with 9106C>T mutation is boxed.

Besides the four MBC cases carrying BRCA2 mutations, genotype analysis was also carried out in six additional unrelated cases enrolled for the presence of FBC in the index case: two with the 9106C>T BRCA2 mutation and four with the IVS16-2A>G BRCA2 mutation. On the whole, genotype analysis was performed in four cases with the 9106C>T BRCA2 mutation, (one family and three isolated cases), and in six IVS16-2A>G BRCA2 mutation cases (three families and three isolated cases). Reconstruction of the chromosome phasing was possible in four families. The analysis carried out in family 25, carrying the 9106C>T BRCA2 mutation, allowed the identification of a common haplotype (5-6-2-6-6) shared by all mutation carriers belonging to this family (Figure 1). This haplotype encompassed a distance of about 3.8 cM between the D13S290 to D13S1695 markers, and was not detected in non-carrier relatives. The isolated cases showed the same allelic pattern for the D13S290, D13S171, and D13S1695 markers, whereas the D13S260 and D13S1698 markers, located within the 3' region flanking the BRCA2 gene, showed an allelic pattern (1,1) different from that of mutation carriers belonging to family 25 (Table 3). Therefore, all isolated cases had the same 5,1,1,6,6 genotype, which is in part consistent with the disease causing haplotype detected in family 25. However, the analysis of 8 internal BRCA2 polymorphisms showed that the same A-C-A-G-T-(-4)-G-C haplotype was present in all the 9106C>T mutation carriers investigated (Table 4). The analysis of 55 healthy donors for 4 of these intragenic polymorphisms (203G>A, IVS8+56C>T, 7470A>G, IVS16-14T>C) showed that, without phase reconstructing, only 15 of them (27%) carried the same haplotype detected in BRCA2 mutation carriers. On these grounds, it is highly unlikely that these four polymorphisms are simultaneously present in the four index cases by chance alone (p = 0.0085, two-tailed Fisher's exact test).

The haplotype analysis by the reconstruction of the chromosome phasing of three IVS16-2A>G BRCA2 mutation families, showed that all mutation carriers had the same 5-4-2 haplotype for the D13S290, D13S171, and D13S1695 markers, whereas all the non-carrier relatives displayed a different allelic pattern for the three markers investigated (Table 5).

## Discussion

MBC accounts for less than 1% of all breast cancers and little is known on the epidemiologic, genetic and clinico-pathologic features of this malignancy [31-34]. Available data indicate that the prevalence of BRCA2 mutations in MBC ranges between 4% and 40% [32-34]. These differences are probably related to the genetic features of the families, with higher rates of BRCA2 mutations in men with a strong family history of cancer, particularly of those belonging to the BRCA2 spectrum.

In our series, the high prevalence of BRCA2 mutation (about 40%) correlated with the high number of relatives with breast cancer present in these families. In fact, excluding from the analysis the two apparently sporadic MBC, the frequency of BRCA2 mutations rises up to 50%. The mean age of breast cancer onset in males with and without BRCA2 mutations is still a controversial issue [16,32,35]. In the present study, we found a median age of diagnosis of 54.5 years for the BRCA2 mutation carriers (range 40–70 years) and of 53.8 years (range 38–62 years) for the w.t. type cases, supporting the findings reported by others [32] indicating that BRCA2 mutation carriers and noncarriers have a similar age at the time of diagnosis.

It has been reported that about 15–20% of MBC patients had a first-degree relative with breast carcinoma, supporting the notion that a positive family history of breast cancer is associated with increased risk of MBC [36,37]. In our survey, all MBC patients carrying deleterious BRCA2 mutations had a family history of breast cancer. This was not due to a selection bias, since the eligibility criteria used for MBC did not require the presence of a positive family history. These findings are in keeping with those reported by others indicating that a positive history of breast cancer is more frequent in BRCA2 gene mutation carriers, with values ranging from 13 to 80% [36-38], although opposite results have been also reported [32]. In our 8 families with a positive history of breast cancer, the presence of a deleterious BRCA2 mutation was not associated with a different occurrence of specific types of cancers as compared to BRCA2-negative families. A significantly higher frequency of breast cancers was observed in the first- and second-degree relatives of mutation carriers, whereas the prevalence of breast cancers in third degree relatives was higher in w.t. families.

Similarly to what reported in other studies [33], also the majority (75%) of our MBC were infiltrating ductal or intraductal carcinomas. One case was classified as intracystic atypic papilloma a relatively infrequent subtype accounting for about 2–5% of all MBC [33]. The patient carrying this tumor was negative to the molecular screening, as those reported in other series [16], ruling out the possibility that BRCA1/2 mutations may confer a significantly increased risk of developing this peculiar MBC histotype.

Available data on the prevalence of BRCA1 and BRCA2 mutations in Italian MBC patients are limited. In a previous study [16], Ottini *et al*. reported one BRCA1 and three BRCA2 mutations including two mutations recurring in central Italy (BRCA1 3345delAG and BRCA2 6696delTC).

The three BRCA2 mutations identified in this study were however different from those we observed. Notably, the MBC cases from our series were characterized by the recurrence of a restricted set of BRCA2 mutations detected in families coming from a limited geographic area (North East of Italy), suggesting the possible existence of a founder effect. Indeed, it has been previously reported that all carriers of the IVS16-2A>G mutation shared a common haplotype, indicating a likely founder effect mainly confined to the Slovenian population [39]. Consistently, our haplotype analysis carried out in six Italian families with the IVS16-2A>G BRCA2 mutation allowed the detection of a common haplotype shared by all mutation carriers. These findings, taken together, support the possible occurrence of a founder effect involving the 5' region flanking the BRCA2 gene in both North-East of Italy and the neighbouring Slovenia, suggesting that the IVS16-2A>G BRCA2 mutations probably recognize a common ancestral origin. The IVS16-2A>G mutation was previously reported by Krajc *et al*. [39] in three breast cancer-only Slovenian families, whereas it was not identified in three families with ovarian cancer, suggesting that the cancer phenotype associated with this mutation is limited to breast cancer. Conversely, our pedigree's analysis of six Italian families with the IVS16-2A>G mutation disclosed the presence of one family in which two ovarian carcinomas were present along with breast cancer, indicating thus that the risk conferred by this mutation is not restricted to breast cancer. Moreover, in the six families with the IVS16-2A>G mutation of our database, two additional MBC were detected (relatives of patients BR195 and BR312), further supporting the increased risk of developing breast cancer in males conferred by this mutation. Since the IVS16-2A>G mutation can result in either a partial or total skipping of exon 17 [29], the increased risk for breast cancer in males due to this mutation may be related to these effects on BRCA2 gene transcription. Studies are under way in our laboratory to elucidate this issue.

With regard to the 9106C>T mutation, the data obtained by haplotype evaluation coupled with the reconstruction of chromosome phasing suggest but do not conclusively prove the occurrence of a possible founder effect also for this mutation. In fact, all the mutation carriers of Family 25 showed the same haplotype for the markers D13S290-D13S260-D13S1698-D13S171-D13S1695; however, the three isolated cases showed a different allelic pattern for the markers D13S260 and D13S1698 located within the 3' region flanking the BRCA2 gene. These results may underlie the occurrence of a possible recombination event involving these two markers in the 3' region flanking the BRCA2 gene as also suggested by the finding that all mutation carriers of Family 25 and the three isolated cases shared the same allelic pattern 6-6 for the 5' markers D13S171 and D13S1695.

In support of this possibility, the analysis of 8 different intragenic BRCA2 gene polymorphisms demonstrated that all carriers of the 9106C>T mutation shared the same haplotype. It can be hypothesized that the common haplotype shared by all mutation carriers could be comprised within a region starting from the 3' end of the gene and extending to the 5' flanking microsatellite markers.

The analysis of larger series of families is however required to conclusively assess whether the 9106C>T mutation is associated with a founder effect in the North East of Italy, enlarging thus the number of founder mutations described for the BRCA2 gene [17,40]. If this will be the case, it would be of interest to verify whether, similarly to what observed for the IVS16-2A>G BRCA2 mutation, also the 9106C>T mutation is associated with a founder effect shared by people from North-East Italy and Slovenia.

## Conclusion

Although carried out on a limited series, the present study confirms that BRCA2 mutations are associated with a significant fraction of breast cancer cases in men. Moreover, the identification of two recurrent BRCA2 mutations predisposing to MBC may be of practical relevance to optimize the molecular analyses aimed at detecting mutation carriers in North East of Italy. In fact, the IVS16-2A>G and the 9106C>T mutations were detected in about 4% of the families we have investigated so far, whereas the recurrent and putative founder mutations account for about 12% of the total. This would support the practical value of starting the mutational analysis of BRCA2 in the North-East Italian population with the search for these particular mutations. This may enhance the collection of larger numbers of families with the same predisposing BRCA2 mutations, providing thus the basis for studies aimed at identifying genetic modifying factors that may modulate the phenotypic manifestations of MBC.

## Competing interests

The author(s) declare that they have no competing interests.

## Authors' contributions

GMM performed haplotype analyses and drafted the manuscript; AVi performed molecular genetic studies and contributed to interpretation of data; MS, and LDP performed BRCA tests and haplotype analyses; CDG, MGT, and AVe collected and analyzed the clinical data; EB performed the statistical analyses; RD was involved in drafting and revising the paper, and participated in design and coordination of the study. All Authors read and approved the final manuscript.

## Pre-publication history

The pre-publication history for this paper can be accessed here:



## Figures and Tables

**Table 1:** Clinicopathologic and genetic characteristics of the 10 MBC cases analyzed for the presence of BRCA1/2 mutations.

**Family**	**Patient**	**Age**	**TNM**	**histology**	**ER**	**PR**	**Method**	**Mutation**	**Effect**	**Affected relatives**
**25**	**BR 22**	40	N.A.	N.A.	N.A.	N.A.	PTT-SSCP	9106C>T	Q2960X	BC(31)s, BC(48)s, BC(44)s, BC(30)s, BC(65)s, BC(37)ptc.
**103**	**BR 111**	59	pT1N0	intraductal carcinoma	N.A.	N.A.	PTT-SSCP MLPA	w.t.		BC(78)s, BC(75)pta, BC(55)ptc, BC(N.A.)ptc, BC(60)ptc, BC(60)ptc, BC(58)ptc, BC(60)ptc.
**125**	**BR 138**	62	pTis	intraductal carcinoma	+	+	PTT-SSCP MLPA	w.t.		BC(82)m.
**127**	**BR 140**	52	pT1cN0	infiltrating ductal carcinoma	+	+	PTT-SSCP MLPA	w.t.		BC(55)mta, BC(47)mta, PC(61)mtu, BC(45)mtc, BCbil.(35)mtc, BC(42)mtc.
**139**	**BR 173**	58	pT2N1biii	infiltrating ductal carcinoma	+	+	PTT-SSCP MLPA	w.t.		/
**140**	**BR 175**	58/66PC	PT4bN0	infiltrating ductal carcinoma	+	+	PTT-SSCP	9106C>T	Q2960X	BCbil.(30)s, BC(40)pta, BC(48)pta.
**161**	**BR 195**	50	N.A.	N.A.	N.A.	N.A.	PTT-SSCP	IVS16-2A>G	exon skipping	BC(38)f, BC(57)pta, PC(N.A.)ptu, BC(50)ptgm, BC(N.A.)ptggf, BC(N.A.)ptga, BC(N.A.)ptga.
**190**	**BR 243**	70	pT1c	infiltrating ductal carcinoma	+	+	PTT-SSCP	IVS16-2A>G	exon skipping	BC(36)d, BC(46)OC(N.A.)m, BC(N.A.)(OC)(N.A.)mta, BC(N.A.)mtc.
**192**	**BR 247**	38	pT4bN1biiiM0	infiltrating ductal carcinoma	+	+	PTT-SSCP MLPA	w.t.		PC(75)f.
**217**	**BR 282**	46	pT1aiiiN0	infiltrating ductal carcinoma	+	+	PTT-Direct Sequencing MLPA	4590C>G	P1491A	BC(N.A.)pta, BC (N.A.)ma.

Number between parentheses indicate the age at diagnosis; N.A.: not available; w.t.: wild-type; PTT: protein truncation test; SSCP: single strand conformation polymorphism; MLPA: multiplex ligation-dependent probe amplification; mt: maternal; pt: paternal; m: mother; f: father; s: sister; d: daughter; gm: grandmother; ggf: great-grandfather; ga: great-aunt; c: cousin; u: uncle; a: aunt. BC: breast cancer; BCbil: bilateral breast cancer; OC: ovarian cancer; PC: prostate cancer.

**Table 2:** Distribution of FBC in relatives of MBC cases belonging to w.t. and BRCA2 mutation carrier families.

	**w.t. families (n = 5)**	**BRCA2 mutation carrier families (n = 4)**		
			
	I+II degree relatives	I+II degree relatives		
	N (%)	N (%)	OR	95% CI
Non-affected	58 (92)	32 (68.1)	1	
Affected	5 (8)	15 (31.9)	5.43	(1.81–16.34)
Total	63	47		

X_1_^2 ^= 10.40; p = 0.001; w.t.: wild-type.

**Table 3:** Genotype analysis of flanking microsatellites associated with the 9106C>T BRCA2 mutation.

	**Markers**						**carrier (+)/non carrier (-)**
	**Probands**	**D13S290**	**D13S260**	**D13S1698**	**D13S171**	**D13S1695**	

**Family 25**							
	BR22	**5**-5	**6**-5	**2**-2	**6**-6	**6**-5	+
	BR23	5-5	5-3	2-5	6-1	5-8	-
	BR99	5-3	2-2	4–6	2-2	5–7	-
	BR100	**5**-4	**6**-2	**2**-4	**6**-2	**6**-5	+
	BR101	**5**-5	**6**-1	**2**-5	**6**-2	**6**-2	+
	BR102	**5**-4	**6**-2	**2**-4	**6**-2	**6**-5	+
	BR103	5-5	5-3	2–5	6-1	5–8	-
**Isolated cases**							
	BR175	**5**,4	**1**,4	**1**,1	**6**,2	**6**,5	+
	BR342	**5**,3	**1**,3	**1**,3	**6**,2	**6**,6	+
	TBL38	**5**,5	**1**,5	**1**,5	**6**,2	**6**,6	+

Numbers in boldface type indicate the 9106C>T mutation alleles. Haplotype segregating with the 9106C>T mutation alleles of phased alleles are separated by a dash. Genotypes of unphased alleles are separated by a comma.

**Table 4:** Haplotype analysis of intragenic BRCA2 polymorphisms associated with the 9106C>T BRCA2 mutation.

**Polymorphisms**		**Family 25**							**Isolated cases**		
**Position**	**Nucleotide change**	**BR22**	**BR23**	**BR99**	**BR100**	**BR101**	**BR102**	**BR103**	**BR175**	**BR342**	**TBL 38**

		M	*w.t*.	*w.t*.	M	M	M	*w.t*.	M	M	M
EX2	203G>A	G>**A**	G-G	G>A	A-**A**	A-**A**	A-**A**	G-G	G>**A**	G>**A**	G>**A**
INTR8	IVS8+56C>T	**C**-C	C-C	C>T	**C**>T	**C**-C	**C**-C	**C**-C	**C**>T	**C**-C	**C**>T
EX10B	1342A>C	**A**>C	C-C	A-A	**A**-A	**A**-A	**A**-A	C-C	**A**-A	**A**-A	**A**-A
EX11-J	3624A>G	A>**G**	A-A	A>G	G-**G**	G-**G**	G-**G**	A-A	A>**G**	A>**G**	A>**G**
EX11K	4035T>C	**T**-T	T-T	T>C	**T**-T	**T**-T	**T**-T	T-T	**T**-T	**T**>C	**T**-T
INTR11Q	IVS11+80del4	**-4**/4	4/4	-4/4	**-4**/-4	**-4**/4	**-4**/-4	4/4	**-4**/4	**-4**/4	**-4**/4
EX14A	7470A>G	A>**G**	A-A	A>G	G-**G**	G-**G**	G-**G**	A-A	A>**G**	A>**G**	A>**G**
INTR17	IVS16-14T>**C**	T>**C**	T>C	T>C	C-**C**	C-**C**	C-**C**	T>C	C-**C**	T>**C**	T>**C**

M: mutated; w.t.: wild-type; numbers in bold indicate the shared allele.

**Table 5:** Genotype analysis of flanking microsatellites associated with the IVS16-2A>G BRCA2 mutation.

		**Markers**			**Carrier (+)/Non carrier (-)**
	
	**Probands**	**D13S290**	**D13S171**	**D13S1695**	
**Family 14**					
	BR6	**5**-5	**4**-2	**2**-5	+
	BR73	5-1	2-2	5–6	-
	BR82	**5**-1	**4**-2	**2**-6	+
**Family 161**					
	BR195	**5**-5	**4**-2	**2**-4	+
	BR285	**5**-5	**4**-2	**2**-4	+
	BR289	5-2	2-1	4–6	-
**Family 190**					
	BR243	**5**-3	**4**-4	**2**-4	+
	BR290	**5**-5	**4**-5	**2**-6	+
	BR291	3–5	4–5	4–5	-
**Isolated cases**					
	BR60	**5**,5	**4**,4	**2**,1	+
	BR85	**5**,5	**4**,2	**2**,5	+
	BR312	**5**,5	**4**,2	**2**,3	+

Numbers in boldface type indicate the IVS16-2A>G mutation alleles. Haplotype segregating with the IVS16-2A>G mutation alleles of phased alleles are separated by a dash. Genotypes of unphased alleles are separated by a comma.
